# Supplementary material for: The interactome of intact mitochondria by cross-linking mass spectrometry provides evidence for coexisting respiratory supercomplexes
Source: Mol Cell Proteomics. 2017 Dec 8;17(2):216–32. doi: 10.1074/mcp.RA117.000470 (PMC5795388; doi:10.1074/mcp.RA117.000470)
Supplement: Supplemental Data [file supp_RA117.000470_133922_0_supp_23978_fzffwf.zip › spectra_annotation/mito_DR_spectra_annotation/499-1-2-1-5-1.pdf]

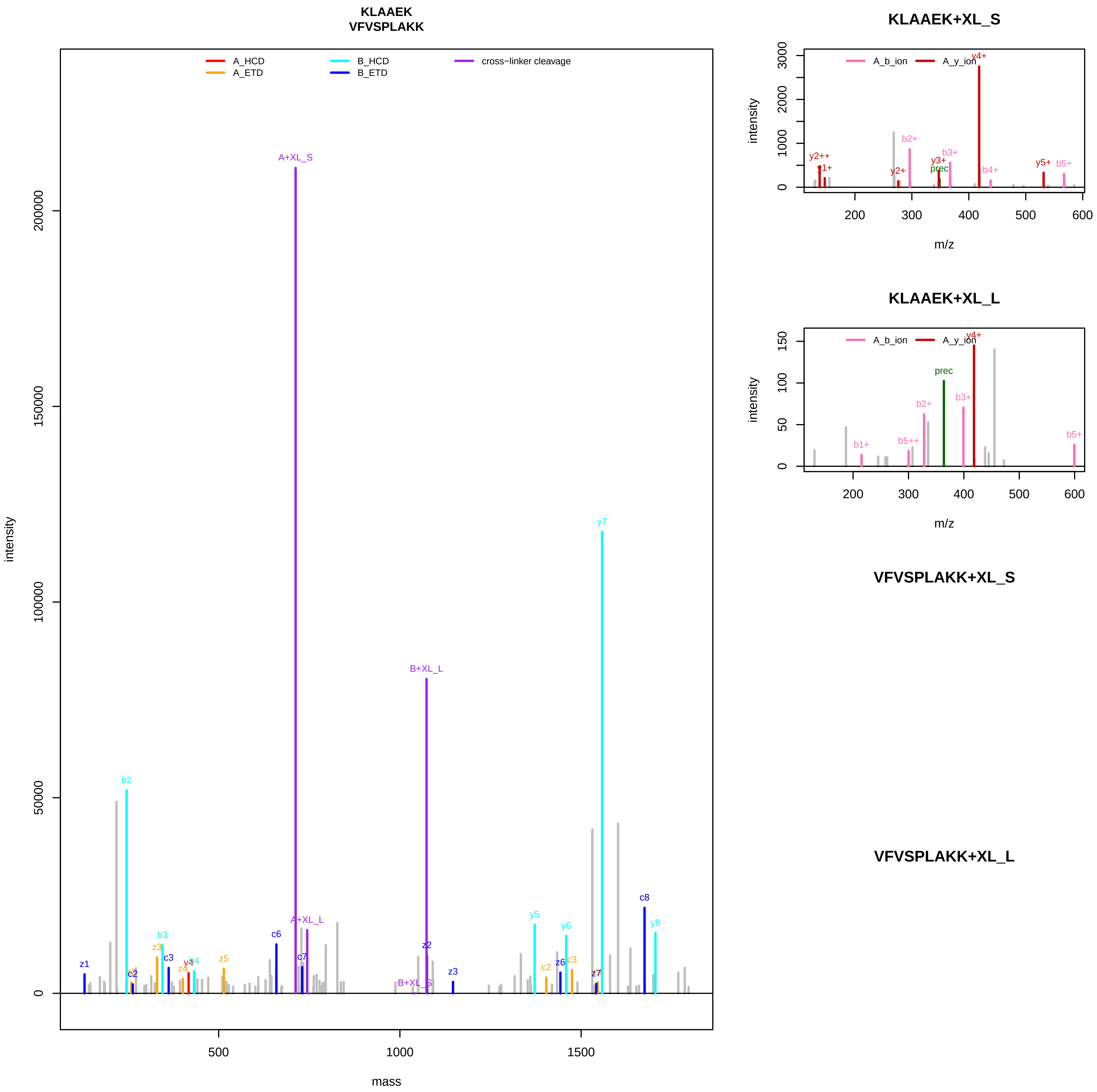
KLAAEK
VFVSPLAKK
A_HCD
A_ETD
B_HCD
B_ETD
cross−linker cleavage
A+XL_S
B+XL_L
A+XL_L
B+XL_S
y7
b2
b3
b4
y4
y5
y6
y8
c2
c3
c6
c7
c8
z1
z2
z3
z4
z5
z6
z7
intensity
mass
0
50000
100000
150000
200000
500
1000
1500
KLAAEK+XL_S
A_b_ion
A_y_ion
y4+
y2++
y1+
y2+
y3+
y5+
b2+
b3+
b4+
b5+
prec
intensity
m/z
0
1000
2000
3000
200
300
400
500
600
KLAAEK+XL_L
A_b_ion
A_y_ion
y4+
prec
b1+
b2+
b3+
b5++
b5+
intensity
m/z
0
50
100
150
200
300
400
500
600
VFVSPLAKK+XL_S
VFVSPLAKK+XL_L
